# A Comparative Evaluation of Clinical Efficacy of Topical Amlexanox and Triamcinolone Acetonide in Oral Lichen Planus

**DOI:** 10.7759/cureus.61242

**Published:** 2024-05-28

**Authors:** Shreya Reddy Chelluri, Raj Kumar Badam, Komali Garlapati, Shaik Ameer, Reshma Priyanka Danam, Divya Harika Pedada

**Affiliations:** 1 Oral Medicine and Radiology, Panineeya Mahavidyalaya Institute of Dental Sciences and Research Centre, Hyderabad, IND

**Keywords:** amlexanox, auto immune, oral lichen planus, thongprasom scale, triamcinolone acetonide

## Abstract

Background: Oral lichen planus (OLP) is a chronic mucocutaneous disease affecting the general population, with its exact etiology remaining unknown. This condition is characterized by T-cell mediated autoimmunity wherein auto-cytotoxic CD8+ T cells precipitate basal cell apoptosis in the oral epithelium. Conventionally, corticosteroids have been the mainstay of treatment for OLP, necessitating the exploration of alternatives to mitigate long-term corticosteroid-related adverse effects. Amlexanox, a topical anti-inflammatory agent, impedes the synthesis and release of histamine, TNF-alpha, and leukotrienes from mast cells, neutrophils, and mononuclear cells, conceivably implicated in OLP pathogenesis.

Aims: The study aims to evaluate and compare the clinical efficacy of topical amlexanox and triamcinolone acetonide in the treatment of OLP.

Objectives: The objectives of this study are (i) to evaluate the lesion size following the topical application of 5% amlexanox paste in the treatment of OLP, (ii) to evaluate the burning sensation of the patient based on the VAS score, and (iii) to compare and evaluate the clinical efficacy of 5% amlexanox with 0.1% triamcinolone acetonide in the treatment of OLP.

Methodology: Forty patients clinically and histopathologically diagnosed with symptomatic OLP were randomly assigned into two groups, each comprising 20 patients. Group A was prescribed topical 5% amlexanox, while Group B received topical 0.1% triamcinolone acetonide with instructions to apply the drug at the site of the lesion intraorally thrice a day after food. The clinical improvement was evaluated using the Thongprasom scale, and the burning sensation was assessed using the visual analog scale (VAS) score weekly over four weeks.

Results: The study showed that there was a statistically significant reduction in the VAS score and size of lesion with each drug individually (p=0.000). There was a statistically significant difference in the mean values of VAS scores and size of the lesion between the first visit and fourth week, indicating a gradual reduction of the burning sensation and size of the lesion in both Group A and Group B, respectively. When both the groups were compared, there was no significant difference (p>0.05) in the reduction of burning sensation between Group A and Group B, indicating that amlexanox was as effective as triamcinolone in reducing the VAS score. However in terms of reduction of lesion size during the second week (p=0.022) and the third week (p=0.013), a statistically significant value was seen with a greater reduction in the size of the lesion in Group B compared to Group A.

Conclusion: Given its anti-inflammatory properties and lower incidence of adverse effects relative to corticosteroids, amlexanox acts as a promising first-line therapeutic option for OLP. In cases of inadequate response, adjunctive therapies can be considered.

## Introduction

Lichen planus (LP) is an autoimmune disease, with its mucosal counterpart known as oral lichen planus (OLP) [1,2]. OLP predominantly affects the buccal mucosa, tongue, and gingiva, with occasional occurrences on the palate, usually presenting bilaterally and involving multiple areas within the oral cavity [1]. Its clinical presentations encompass various forms, including reticular, papular, erythematous, erosive, plaque-like, and bullous manifestations. The prevalence of OLP is estimated to be between 1 and 2 percent [2]. While both genders can be affected, it primarily impacts middle-aged women, with a female-to-male ratio of 1.4:1 [1]. The etiology of OLP is multifactorial involving factors such as stress, anxiety, depression, genetic predisposition, systemic diseases, chronic liver disease, and the presence of the Hepatitis C virus [2]. 

OLP is characterized by CD-8 (cluster differentiation) cell-induced damage to basal keratinocytes, triggering apoptosis. This process is further complicated by increased cytokine levels, recruitment of Langerhans cells, and clonal expansion of cytotoxic cells, contributing to its pathogenesis. Additionally, non-specific mechanisms like mast cell degranulation and MMP-1 activation exacerbate T-cell accumulation, basement membrane disruption, and keratinocyte apoptosis [2].

Despite the availability of various treatment modalities, managing OLP remains a significant clinical challenge due to its chronic nature, high recurrence rates, and potential adverse effects associated with long-term medication use. Triamcinolone acetonide, a commonly used topical corticosteroid, has effectively reduced inflammation and alleviated symptoms associated with OLP. However, concerns regarding its safety profile with prolonged use necessitate the exploration of alternative treatment options.

Objective

The study aims to evaluate and compare the clinical efficacy of topical amlexanox and triamcinolone acetonide in treating OLP by assessment of the lesion size and symptom relief with both treatments. The study intends to provide evidence-based recommendations for selecting topical agents for managing OLP.

## Materials and methods

A randomized, positive-controlled clinical study was conducted on patients between the ages of 18 and 70 years reporting to the Department of Oral Medicine & Radiology, Panineeya Mahavidyalaya Institute of Dental Sciences and Research Centre, Hyderabad, who were clinically and histopathologically diagnosed with symptomatic OLP. The study protocol received approval from the Institutional Ethical Review Board (Ethical clearance number: PMVIDS&RC/IEC/OMR/DN/0315-19). The study was conducted in accordance with the Declaration of Helsinki, the code of ethics of the World Medical Association, and all participants provided consent and the study was conducted maintaining confidentiality.

Patients with any other mucosal disease or skin disease that may be associated with oral lesions, patients who were on medication - either topical or systemic - for oral mucosal lesions, patients with known allergies or contraindications to study medications, patients with a known history of systemic diseases or conditions where steroids are contraindicated, and pregnant women were excluded from the study.

The subjects were explained in detail about the study, and written informed consent was obtained from the patient. The detailed history of the patient and extraoral and intraoral findings were recorded in the case history proforma.

**Figure 1:**
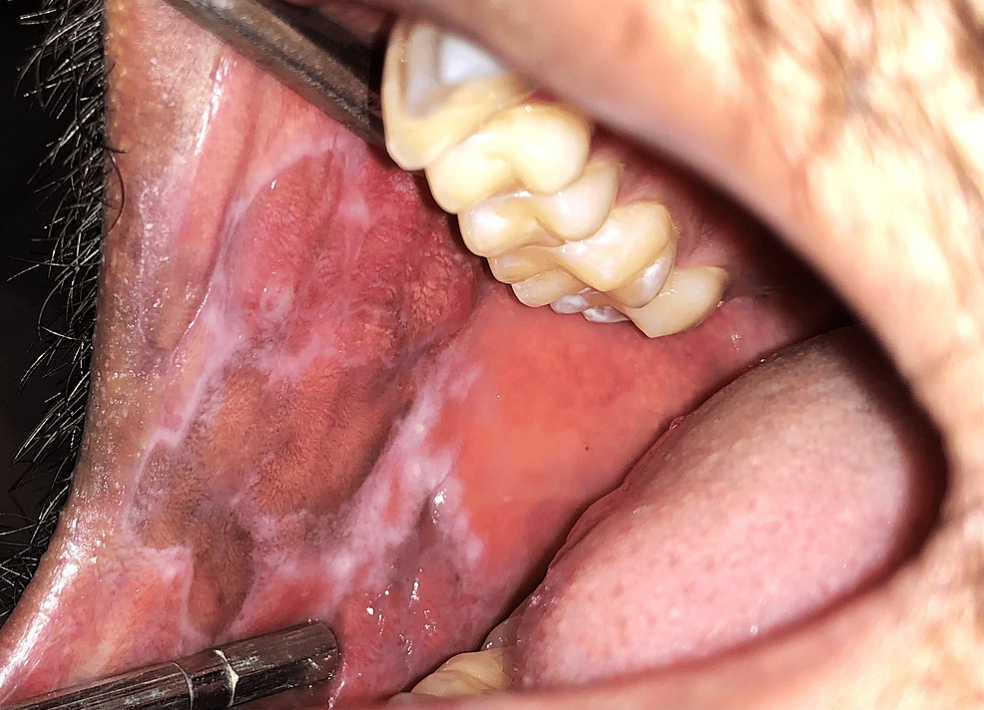
Pre-treatment image of erosive lichen planus affecting the right buccal mucosa during the initial visit (Group A)

**Figure 2:**
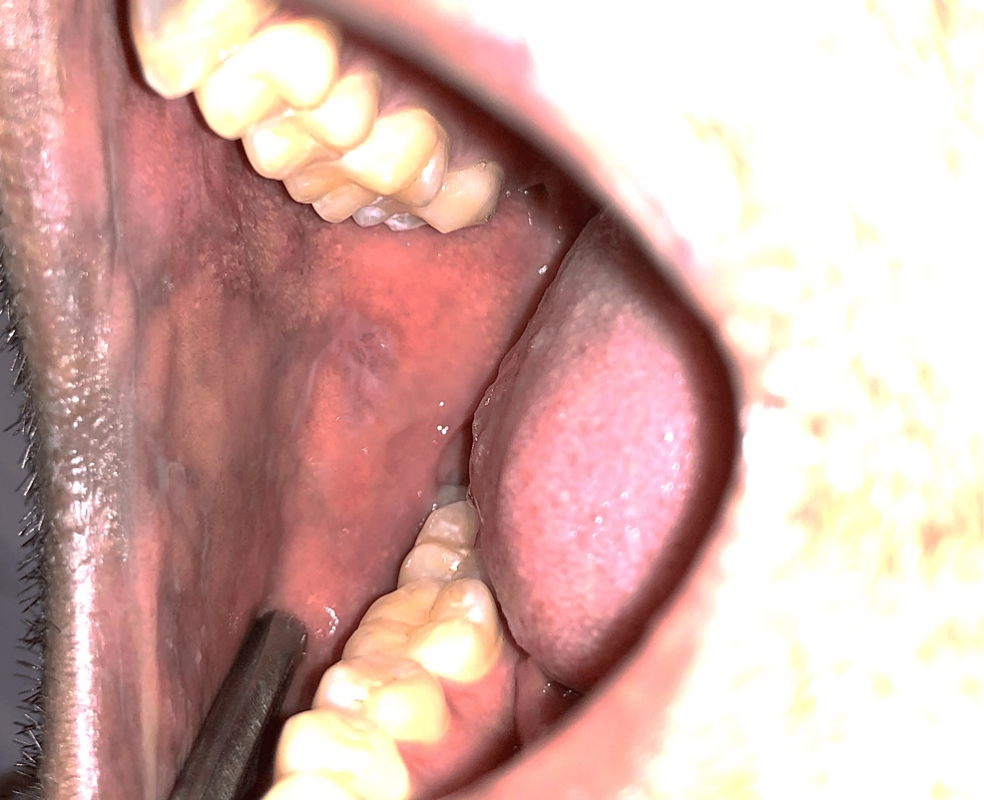
Post-treatment image of reduction of erosive lichen planus affecting the right buccal mucosa (Group A)

**Figure 3:**
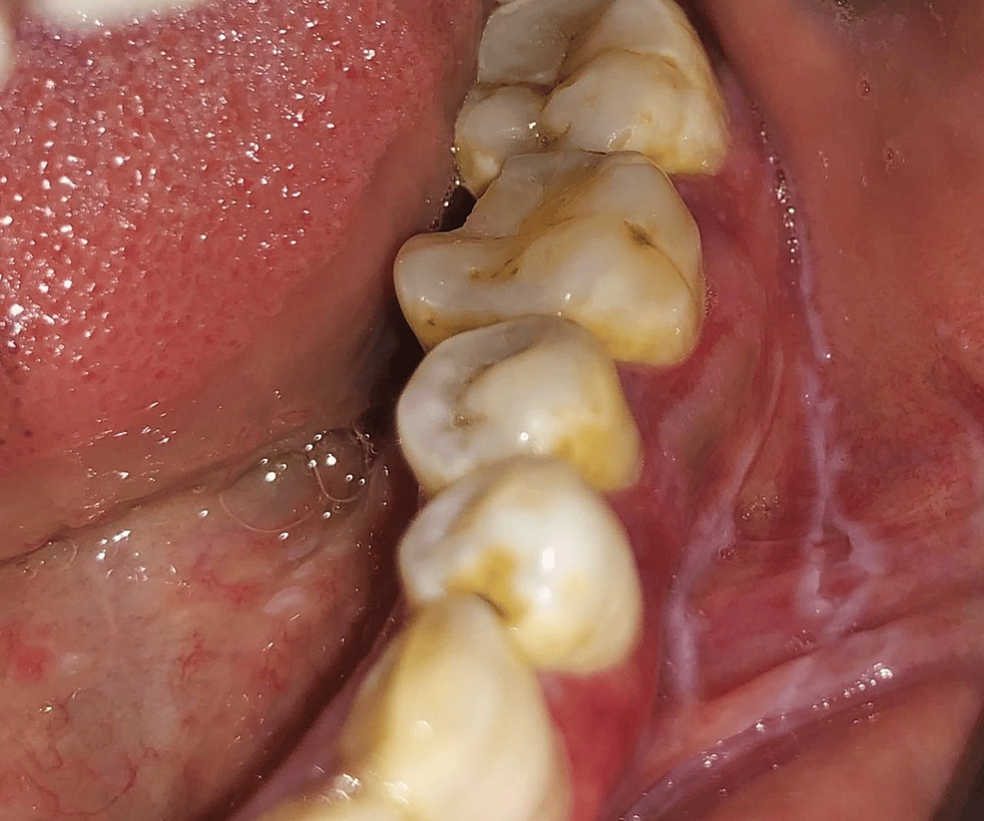
Pre-treatment image of erosive lichen planus affecting the gingival margin and lower buccal vestibule during the initial visit (Group B)

**Figure 4:**
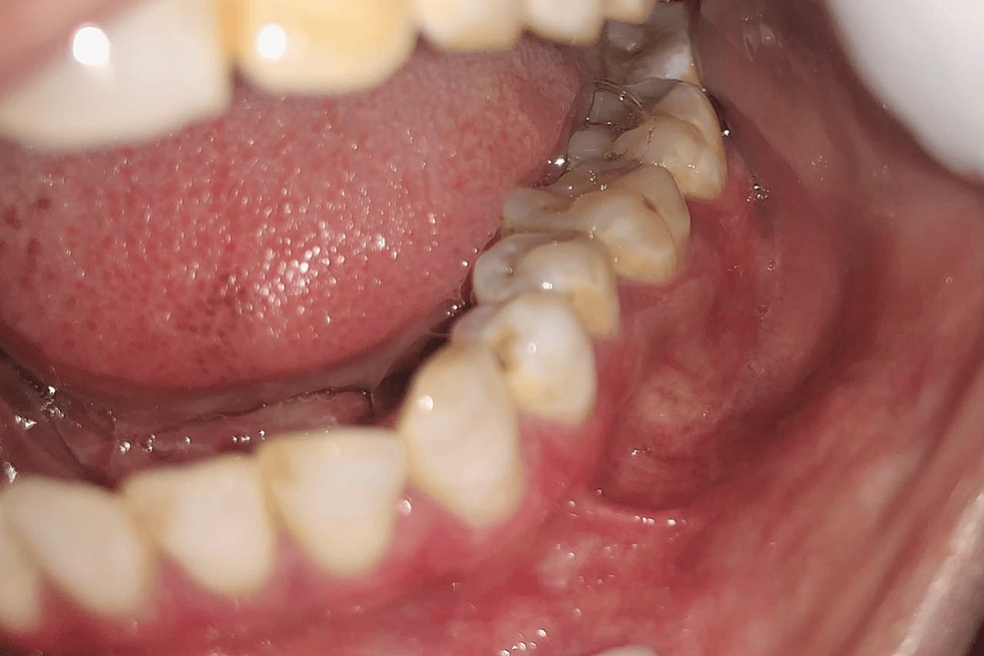
Post-treatment image of reduction of erosive lichen planus affecting gingival margin and the lower buccal vestibule (Group B)

**Figure 5:**
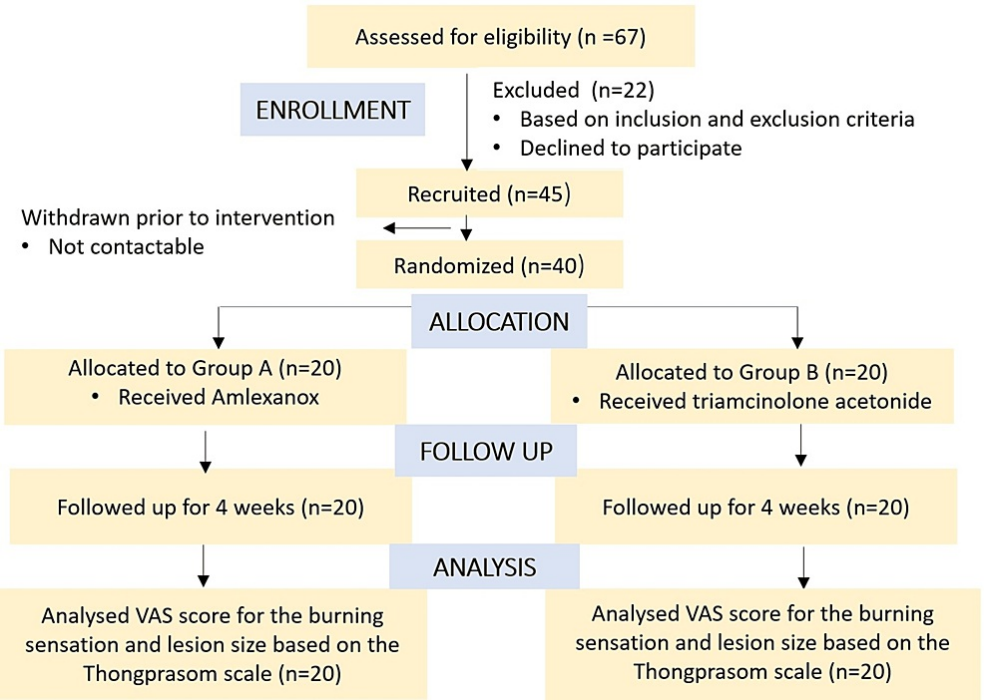
CONSORT diagram VAS: Visual analog scale

Forty subjects were included in the present study and were randomly divided into two groups, A and B, with 20 patients in each group. A randomized sampling method was used, specifically centralized randomization, with the department administrative officer ensuring that the allocation sequence remained concealed until the interventions were assigned. Group A patients were prescribed topical 5% Amlexanox (Figure 1 and Figure 2), and Group B patients were prescribed topical 0.1% Triamcinolone acetonide (Figure 3 and Figure 4) thrice a day after food. The total duration of the treatment was four weeks, starting from the initial visit. The flow of participants through each stage of the trial is detailed in the CONSORT diagram (Figure 5).

For both groups, lesion size was measured using the Thongprasom scale [3]. The scoring included the following: 0 for no lesion, 1 for mild white striae without an erythematous area, 2 for white striae with an atrophic area of less than 1 cm^2^, 3 for white striae with an atrophic area of more than 1 cm^2^, 4 for white striae with an erosive area less than 1 cm^2^, and 5 for white striae with an erosive area of more than 1 cm^2^ and burning sensation was assessed by the visual analog scale (VAS) [3] at the initial visit and weekly visits till the cessation of treatment. This scale consists of a 10-cm horizontal line, where 0 represents no pain and 10 represents extreme pain. Patients marked the point on the line to indicate their pain perception.

Statistical analysis

The data analysis was conducted using IBM SPSS Statistics for Windows, Version 20 (Released 2011; IBM Corp., Armonk, New York, United States). The one-way analysis of variance (ANOVA) and an independent-sample t-test were utilized to analyze recordings of the VAS score for the burning sensation and lesion size based on the Thongprasom scale. A p-value below 0.05 was deemed to be statistically significant.

## Results

Gender and age distribution

Descriptive statistics were used to analyze gender and age distribution. There were 32 females and eight males in Groups A and B, making up 80% females and 20% males, indicating female predilection (Table 1). In both groups, the age of the subjects ranged from 19-65 years, and the overall mean age of the subjects included was 44.3 years (Table 2). The data showed that OLP is most observed in the middle-aged group in this study.

**Table 1:** Gender distribution of subjects in two groups

Distribution of Participants	Sex	Number (%)
Group-A 5% Amlexanox	Male	4(20)
	Female	16(80)
Group-B 0.1% Triamcinolone acetonide	Male	4(20)
	Female	16(80)
Total	Male	8(20)
	Female	32(80)

**Table 2:** Age distribution of subjects in two groups

Distribution of Participants	Sex	Number (%)	Mean age	Std. Deviation	Std. Error Mean
Group-A 5% Amlexanox	Male	4(20)	40.75	18.733	9.366
	Female	16(80)	41.25	8.021	2.005
Group-B 0.1% Triamcinolone acetonide	Male	4(20)	46.00	11.343	5.672
	Female	16(80)	49.31	12.642	3.161

Type and site distribution

Descriptive statistics were used to analyze the distribution of LP types and lesion locations. In the current study, 52.5% of patients in the current study had erosive LP, 45% had atrophic LP, and 2.5% had bullous LP (Table 3). As a result, most cases seen in this study were erosive, followed by atrophic, and finally bullous. The majority of the subjects in both groups had the lesion on the buccal mucosa, accounting for 47.5%, followed by buccal mucosa and gingiva with 20%, then 12.5% on buccal mucosa and tongue, 7.5% involving multiple sites, 5% only on the tongue, 5% only on gingiva, and 2.5% on gingiva and tongue together (Table 4). 

**Table 3:** Distribution of clinical types of lichen planus in current study groups

Clinical variants	Sex	Number (%)	Total (%)
Bullous	Male	0(0)	1(2.5)
	Female	1(3.125)	
Erosive	Male	4(50)	21(52.5)
	Female	17(53.12)	
Atrophic	Male	4(50)	18(45)
	Female	14(43.75)	
All variants	Male	8(100)	40(100)
	Female	32(100)	

**Table 4:** Distribution of lesion sites in current study groups

Lesion site	Sex	Number%	Total (%)
Buccal mucosa	Male	4(50)	19(47.5)
	Female	15(46.8)	
Buccal mucosa and tongue	Male	1(12.5)	5(12.5)
	Female	4(12.5)	
Buccal mucosa and gingiva	Male	1(12.5)	8(20)
	Female	7(21.87)	
Tongue	Male	1(12.5)	2(5)
	Female	1(3.125)	
Multiple	Male	0(0)	3(7.5)
	Female	3(9.37)	
Gingiva	Male	1(12.5)	2(5)
	Female	1(3.125)	
Gingiva and tongue	Male	0(0)	1(2.5)
	Female	1(3.125)	
All lesion sites	Male	8(100)	40(100)
	Female	32(100)	

Evaluation of burning sensation measured by the VAS

Group A

**Figure 6:**
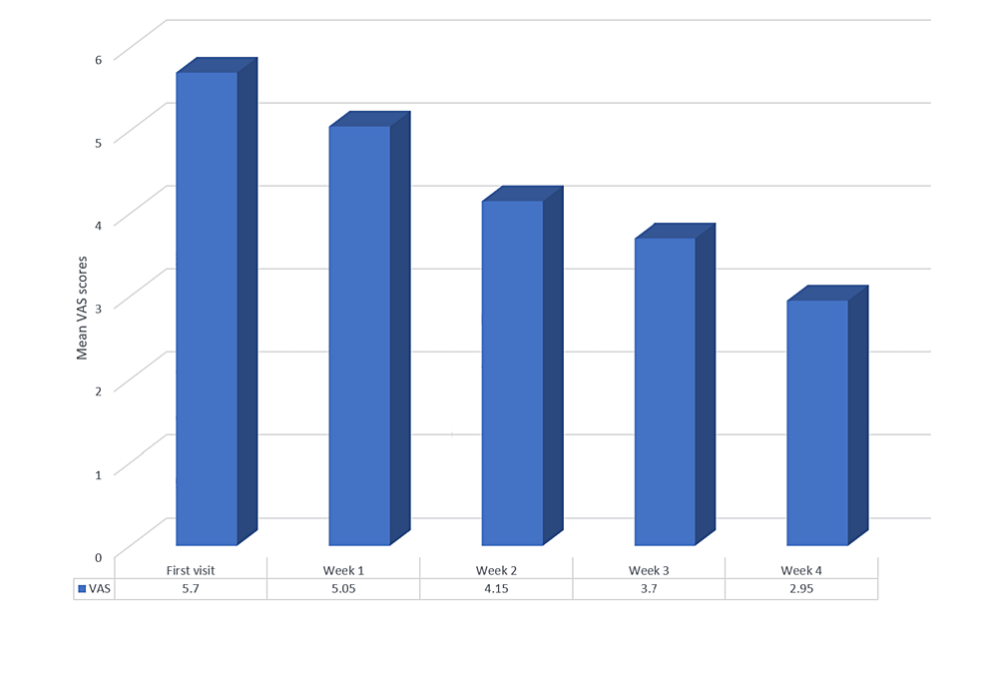
Mean VAS scores across four weeks in Group A (5% amlexanox) VAS: Visual analog scale

The one-way ANOVA showed a significant reduction in the mean VAS score from 5.70 during the first visit to 2.95 at the end of the fourth week (p<0.05). This indicates a significant difference in mean VAS scores between patient visits at different time intervals. (Table 5 and Figure 6). 

**Table 5:** ANOVA comparing mean VAS scores across four weeks of treatment in Group A (5% amlexanox) p ≤0.05 considered statistically significant The asterisk (*) indicates statistically significant findings VAS: Visual analog scale

Schedule	Number(%)	Mean	Std. Deviation	Std. Error	95% CI		Minimum	Maximum	p-value
					Lower	Upper			
First visit	20(100)	5.70	1.750	.391	4.88	6.52	3	9	0.000*
Week 1	20(100)	5.05	1.669	.373	4.27	5.83	3	8	
Week 2	20(100)	4.15	1.725	.386	3.34	4.96	2	7	
Week 3	20(100)	3.70	1.922	.430	2.80	4.60	1	7	
Week 4	20(100)	2.95	2.502	.559	1.78	4.12	0	7	
Total	100(100)	4.31	2.135	.214	3.89	4.73	0	9	

Group B

**Figure 7:**
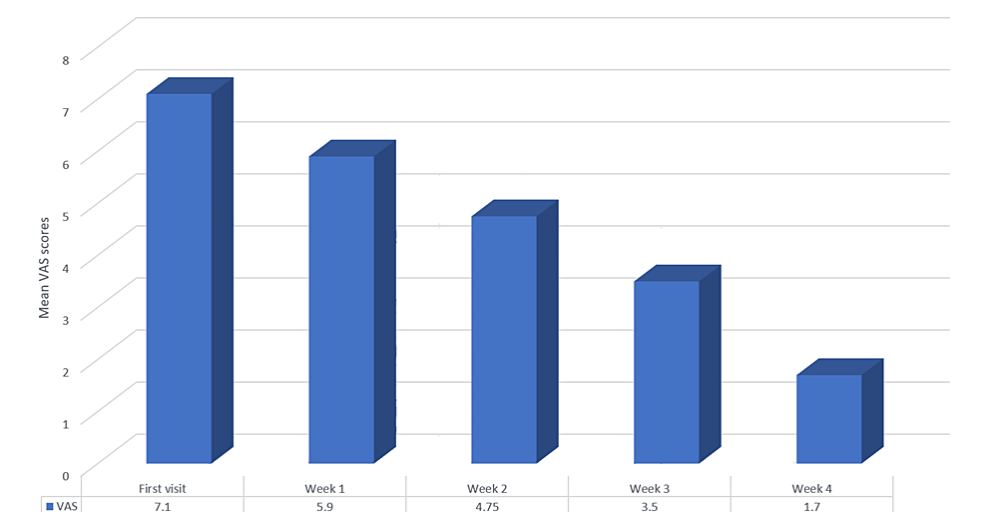
Mean VAS scores across four weeks in Group B (0.1% triamcinolone acetonide) VAS: Visual analog scale

The one-way ANOVA was used to determine significant differences between mean VAS scores at different time intervals. The initial mean VAS was 7.10, dropping to 1.75 at the end of week 4, with p=0.000, indicating a significant difference in mean VAS scores between patient visits (Table 6 and Figure 7). 

**Table 6:** ANOVA comparing mean VAS scores across four weeks of treatment in Group B (0.1% triamcinolone) p ≤0.05 considered statistically significant The asterisk (*) indicates statistically significant findings VAS: Visual analog scale

Schedule	Number(%)	Mean	Std. Deviation	Std. Error	95% CI		Minimum	Maximum	p-value
					Lower	Upper			
First visit	20(100)	7.10	1.553	.347	6.37	7.83	4	10	0.000*
Week 1	20(100)	5.90	1.447	.324	5.22	6.58	3	9	
Week 2	20(100)	4.75	1.410	.315	4.09	5.41	2	8	
Week 3	20(100)	3.50	1.469	.328	2.81	4.19	2	7	
Week 4	20(100)	1.70	1.750	.391	.88	2.52	0	6	
Total	100(100)	4.59	2.408	.241	4.11	5.07	0	10	

Evaluation of the size of the lesion by the Thongprasom scale

Group A

**Figure 8:**
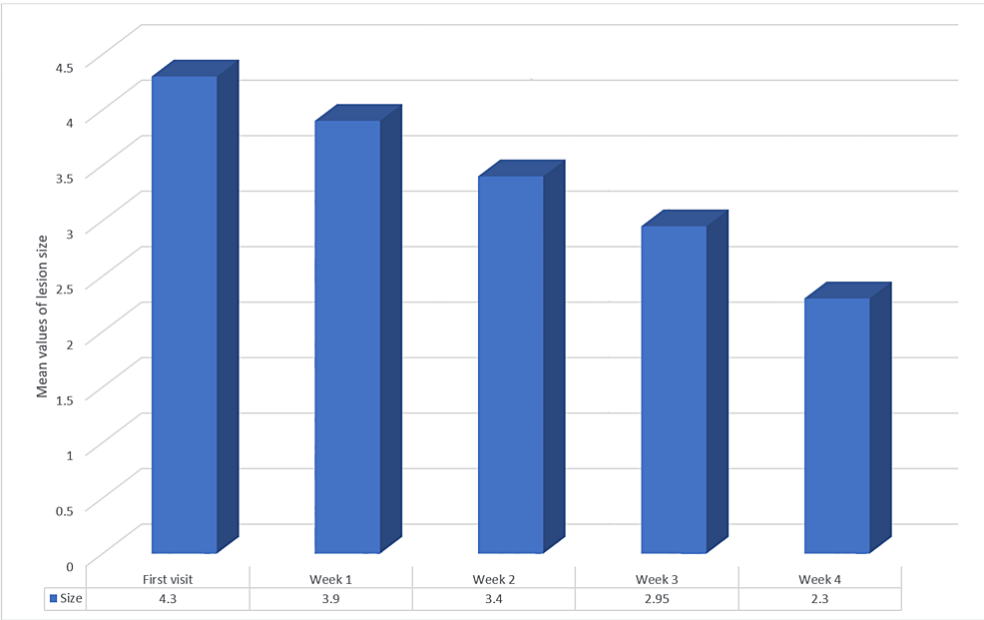
Mean values of the lesion size across four weeks in Group B (5% amlexanox)

The one-way ANOVA test found significant differences in the lesion size measured by the Thongprasom scale at different time intervals. The lesion size reduced from a mean value of 4.30 to 2.30 by the end of the fourth week, with p=0.000, indicating statistical significance (Table 7 and Figure 8). 

**Table 7:** ANOVA comparing the mean value of the lesion size across four weeks in Group A (5% amlexanox) p ≤0.05 considered statistically significant The asterisk (*) indicates statistically significant findings

Schedule	Number (%)	Mean	Std. Deviation	Std. Error	95% CI		Minimum	Maximum	p-value
					Lower	Upper			
First visit	20(100)	4.30	.865	.193	3.90	4.70	3	5	0.000*
Week 1	20(100)	3.90	.912	.204	3.47	4.33	2	5	
Week 2	20(100)	3.40	1.095	.245	2.89	3.91	2	5	
Week 3	20(100)	2.95	1.317	.294	2.33	3.57	1	5	
Week 4	20(100)	2.30	1.490	.333	1.60	3.00	0	5	
Total	100(100)	3.37	1.338	.134	3.10	3.64	0	5	

Group B

**Figure 9:**
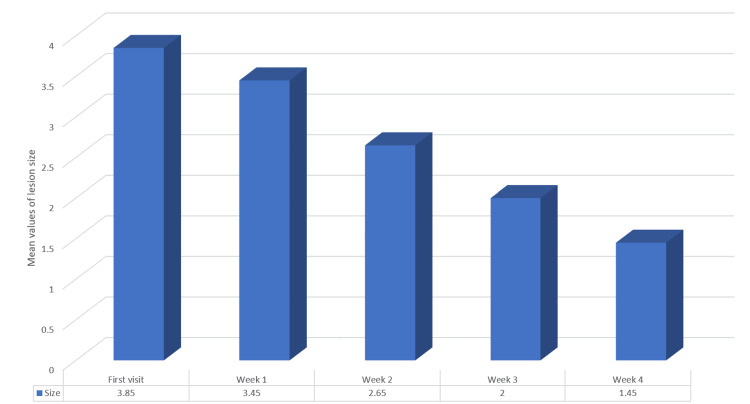
Mean values of the lesion size across four weeks in Group B (0.1% triamcinolone acetonide)

The one-way ANOVA showed a statistically significant difference between mean sizes. The mean size reduced from 3.85 to 1.45 by the fourth week (p=0.000) (Table 8 and Figure 9). 

**Table 8:** ANOVA comparing the mean value of the lesion size across four weeks in Group A (0.1% triamcinolone acetonide) p ≤0.05 considered statistically significant The asterisk (*) indicates statistically significant findings

Schedule	Number(%)	Mean	Std. Deviation	Std. Error	95% CI		Minimum	Maximum	p-value
					Lower	Upper			
First visit	20(100)	3.85	.988	.221	3.39	4.31	2	5	0.000*
Week 1	20(100)	3.45	.826	.185	3.06	3.84	2	5	
Week 2	20(100)	2.65	.875	.196	2.24	3.06	1	4	
Week 3	20(100)	2.00	.973	.218	1.54	2.46	1	4	
Week 4	20(100)	1.45	1.146	.256	.91	1.99	0	4	
Total	100(100)	2.68	1.302	.130	2.42	2.94	0	5	

Comparison between Group A and Group B at different time intervals using an independent t-test

Comparison of the VAS Score Between Group A and Group B

**Figure 10:**
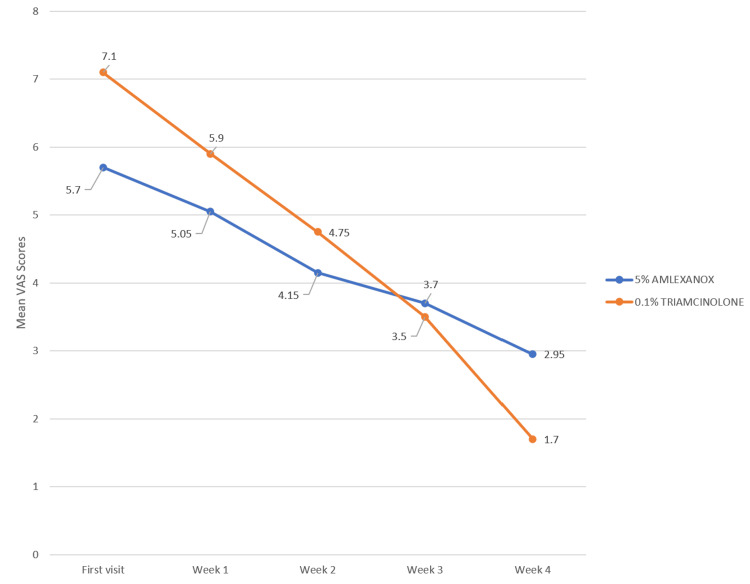
Graphical representation comparing the mean VAS scores between Group A and Group B VAS: Visual analog scale

The independent t-test results confirmed that there was no significant difference in the reduction of burning sensation between the amlexanox and triamcinolone-treated groups. The p-values for the first, second, and third weeks were 0.093, 0.236, and 0.714, respectively, and were not statistically significant. Similarly, the p-value for the fourth week was 0.075, which was also not statistically significant. These results strongly suggest that amlexanox is a viable alternative to triamcinolone for treating burning sensation. Hence, amlexanox was as effective as triamcinolone in reducing the VAS score (Table 9 and Figure 10).

**Table 9:** Mean comparison of the VAS score between Group A and Group B p ≤0.05 considered statistically significant The asterisk (*) indicates statistically significant findings VAS: Visual analog scale

Schedule	Group	Number (%)	Mean	Std. Deviation	Std. Error Mean	Mean difference	t(df)	p-value
First Visit	A	20(100)	5.70	1.750	.391	-1.400	-2.676 (38)	0.011*
	B	20(100)	7.10	1.553	.347			
First Week	A	20(100)	5.05	1.669	0.373	-0.850	-1.720 (38)	0.093
	B	20(100)	5.90	1.447	0.324			
Second Week	A	20(100)	4.15	1.725	0.386	-0.600	-1.204 (38)	0.236
	B	20(100)	4.75	1.410	0.315			
Third Week	A	20(100)	3.70	1.922	0.430	0.200	0.370 (38)	0.714
	B	20(100)	3.50	1.469	0.328			
Fourth Week	A	20(100)	2.95	2.502	0.559	1.250	1.831 (38)	0.075
	B	20(100)	1.70	1.750	0.391			

Comparison of the Size of Lesions Between Group A and Group B 

The independent t-test was used to compare triamcinolone and amlexanox for reducing the lesion size over four weeks. During the first week, there was no significant difference (p=0.110). During the second and third weeks, triamcinolone showed a statistically significant difference in lesion reduction compared to amlexanox (p=0.022 and p=0.013, respectively). By the end of the fourth week, there was no significant difference between the two groups again (p=0.300). Therefore, the study concluded that Triamcinolone was more effective during the second and third weeks (Table 10).

**Table 10:** Mean comparison of the size of lesions between Group A and Group B p ≤0.05 considered statistically significant The asterisk (*) indicates statistically significant findings

Schedule	Group	Number(%)	Mean	Std. Deviation	Std. Error Mean	Mean difference	t(df)	p-value
First Visit	A	20(100)	4.30	0.865	0.193	0.450	1.533 (38)	0.134
	B	20(100)	3.85	0.988	0.221			
First Week	A	20(100)	3.90	1.669	0.912	0.450	1.636 (38)	0.110
	B	20(100)	3.45	1.447	0.826			
Second Week	A	20(100)	3.40	1.095	0.245	0.750	2.392 (38)	0.022*
	B	20(100)	2.65	0.875	0.196			
Third Week	A	20(100)	2.95	1.317	0.294	0.950	2.594 (38)	0.013*
	B	20(100)	2.00	0.973	0.218			
Fourth Week	A	20(100)	2.30	1.490	0.333	0.850	2.022 (38)	0.051
	B	20(100)	1.45	1.146	0.256			

## Discussion

In the treatment of OLP, achieving complete remission remains challenging, and the approach has been primarily symptomatic. To date, topical corticosteroids remain the front-line therapy for OLP, owing to their capacity to regulate both inflammatory and immune responses. Nevertheless, prolonged use of these agents may precipitate localized adverse effects, including oral mucosal thinning, application discomfort, and heightened infection vulnerability.

To minimize steroid usage, various steroid-sparing agents such as immunosuppressant drugs (such as cyclosporine and tacrolimus), retinoids, dapsone, griseofulvin, hydroxychloroquine sulfate, and phenytoin have been explored. Additionally, non-pharmacologic modalities like photodynamic therapy, UV radiation, laser treatments, and psychiatric therapy offer alternative approaches. Natural remedies such as lycopene, curcumin, green tea, and aloe vera also present viable options for OLP treatment. Furthermore, emerging agents like topical amitriptyline, amlexanox, bacillus Calmette-Guerin polysaccharide nucleic acid (BCGPSN), hyaluronic acid, and topical thalidomide have shown promise in recent studies [4,5].

Amlexanox has its efficacy proven in treating recurrent aphthous ulcers due to its remarkable anti-inflammatory properties, but studies regarding its role in suppressing inflammation in OLP are limited. [6,7] Chalkoo et al. [6] and Shrivastava et al. [7] compared the clinical efficacy of 0.1% topical triamcinolone acetonide and 5% amlexanox in recurrent aphthous stomatitis. Their findings suggest that amlexanox oral paste exhibits superior outcomes in decreasing erythema, ulcer size, pain, and healing time of recurrent aphthous ulcers. As a result, they proposed amelexanox as a viable substitute for topical corticosteroids. However, in the literature, only a single study conducted by Fu et al. [3] was identified comparing the efficacy of amlexanox vs. dexamethasone in the treatment of OLP. Amlexanox is a topical anti-inflammatory drug that suppresses the synthesis and secretion of histamine, TNF-alpha, and leukotrienes from various immune cells, such as mast cells, neutrophils, and mononuclear cells. This suppression mechanism likely involves an elevation in the levels of cyclic adenosine monophosphate within inflammatory cells [5].

OLP involves different locations in oral mucosa, and several authors reported studies regarding the occurrence of these lesions at various sites in the oral cavity. In the present study in both Group A and Group B, the majority of the subjects had the site of occurrence of the lesion on buccal mucosa, accounting for 47.5%, followed by buccal mucosa and gingiva with 20% then 12.5% on buccal mucosa and tongue,7.5% involving multiple sites, 5% only on the tongue, 5% only on gingiva and 2.5% on gingiva and tongue together. Hence, buccal mucosa is the most commonly involved site in the present study. Similarly, the studies conducted by Mousavi et al. [8], Sonthalia et al. [9], Budimir et al. [10], Kia et al. [11], Tak and Chalkoo [12], Mostafa and Ahmed [13], Bandyopadhyay et al. [14], Girija et al. [15] Joshy et al. [16], Kaomongkolgit et al. [17], and Chalkoo et al. [18] where buccal mucosa is the most common site involved in OLP.

OLP has the potential to impact individuals of any gender, although it tends to occur more commonly in females than males. Some research suggests either a male prevalence or an equal likelihood of occurrence across both genders. In the present study, it was seen that females were more affected than males. Similarly, the studies by Silverman et al. [19], Brown et al. [20], Arora et al. [21], Tak et al. [12], Veerabasvaiah et al. [22], Thomas et al. [23], Girija et al. [15], Kaomongkolgit et al. [17], Chalkoo et al. [18], and Fu et al. [3] showed female predilection. However, studies by Munde et al. [24] and Bandyopadhyay et al. [14] reported an increased incidence of OLP in males.

Age distribution of participants in studies on LP has been a subject of interest, reflecting potential demographic trends and variations in disease presentation. In the present study, both Group A and Group B exhibited a broad age range spanning from 19 to 65 years. For Group A, the mean age was 41.25 for females and 40.75 for males, while for Group B, it was 49.31 for females and 46 for males. The overall mean age across both groups was 44.3 years. This aligns with findings in studies by Ingafou et al. [25], Arora et al. [21], Tak et al. [12], Girija et al. [15], and Joshy et al. [16]. Notably, our mean age is slightly lower than that in studies by Silverman et al. [19], Brown et al. [20], Fu et al. [3], Mostafa et al. [13], and Kaomongkolgit et al. [17], while it is higher than that in the study by Munde et al. [24].

The incidence of each clinical type in OLP has differed in various studies described in the literature. The present study enrolled participants diagnosed with symptomatic OLP, encompassing all clinical variations of the condition, contrary to the study by Fu et al. [3], which specifically focused on erosive cases. In the present study, erosive LP was diagnosed in 55 % of patients in Group A, followed by atrophic LP in 45 % of patients and bullous LP in 5% of patients. Erosive LP was diagnosed in half of the individuals in Group B, while atrophic LP was detected in the other half. In both groups, 52.5% of patients had erosive LP, 45 % had atrophic LP, and 2.5% had bullous LP. The reticular pattern of OLP is frequently asymptomatic and does not require intervention. As a result, the majority of cases seen in this study were of the erosive type of LP, which aligns with the studies conducted by Sonthalia et al. [9], Irani et al. [26], and Mansourian et al. [27] where the erosive form was the most common clinical type. However, the studies by Tak et al. [12], Chitturi et al. [28], Veerabasvaiah et al. [22], Bandyopadhyay et al. [14], Chalkoo et al. [5] reported that reticular form was the commonest form noted in these studies and also the study by Kaomongkolgit et al. [17] stated atrophic form to be seen more predominantly.

The present study examines the efficacy of treatments amlexanox and triamcinolone acetonide, utilizing the Thongprasom scale and VAS to assess clinical improvement for four weeks. In contrast, the study by Fu et al. [3] was confined to a seven-day period. None of the patients in the present study reported any discomfort throughout the treatment. However, Fu et al. [3] found discomfort in the amlexanox group, three patients in the topical amlexanox-treated group complained about a little burning, dry mouth sensations, and bleeding when applying the medicine.

In the current study, intra-group assessments revealed a significant reduction in burning sensation and lesion size in both the amlexanox and triamcinolone groups over the four-week period. Amlexanox demonstrated a gradual decline in VAS scores from the first visit, with statistical significance observed from the third week to fourth week. The mean VAS score dropped from 5.70 (standard deviation = 1.750) at the first visit to 2.95 (standard deviation = 2.502) by the end of the fourth week. This finding aligns with Fu et al. [3], who also reported significant reductions in mean VAS scores with amlexanox treatment. Similarly, the triamcinolone acetonide group exhibited a significant reduction in burning sensation, with the mean VAS score decreasing from 7.10 (standard deviation = 1.553) at baseline to 1.70 (standard deviation = 1.750) by the fourth week. This result corroborates findings from studies by Thomas et al. [23] and Joshy et al. [16], all of which reported effective pain and burning sensation reduction in OLP patients using 0.1% triamcinolone acetonide.

When comparing both groups in the present study, there was no significant difference in the reduction of burning sensation between the amlexanox and triamcinolone treatments from the first to the fourth week, suggesting that both drugs are equally effective. This is consistent with Fu et al. [3], who found no significant differences between amlexanox and topical dexamethasone in alleviating this symptom. In the present study, both treatments effectively reduced lesion size, but Triamcinolone exhibited a greater reduction during the second and third weeks compared to amlexanox. This finding contrasts with the study by Fu et al. [3], where no difference was observed between amlexanox and topical steroid groups in reducing the erosive area.

Strengths of the study

The study provided valuable insights into the relative efficacy and safety profiles of amlexanox and triamcinolone acetonide. It also explored demographic and clinical variations in OLP, aiding in the development of tailored treatment approaches for diverse patient groups.

Limitations of the study

The limitations of the present study include its relatively small sample size, which may limit the generalizability of findings. Additionally, the study could have further explored potential confounding variables, such as patient comorbidities or concomitant medications, which might have impacted treatment outcomes. The observed variations in the study emphasize the need for further research in treating LP and exploring amlexnox’s potential role in the management. Future research in OLP should focus on conducting large-scale randomized controlled trials with longer follow-up periods to evaluate the durability of treatment responses and recurrence rates.

## Conclusions

In this randomized clinical study of assessing and comparing the clinical efficacy of topical amlexanox and triamcinolone acetonide in treating OLP, both treatments demonstrated significant clinical improvement over the four-week duration. Both treatment groups saw a reduction in burning sensation and lesion size, with no significant difference between amlexanox and triamcinolone in reducing burning sensation. However, triamcinolone showed a greater reduction in lesion size during the second and third weeks compared to Amlexanox. These findings suggest that amlexanox could be considered an effective alternative to triamcinolone in managing OLP, especially for patients with concerns regarding the prolonged use of corticosteroids.

The study also provided valuable insights into the demographic and clinical variations of OLP, highlighting the predominance of erosive LP and the higher prevalence among middle-aged females. Such insights contribute to the development of tailored treatment approaches for diverse patient groups. While the study sheds light on the relative efficacy of amlexanox and triamcinolone, it is essential to acknowledge its limitations, including the relatively small sample size and the need for further exploration of potential confounding variables. Future research should focus on conducting large-scale randomized controlled trials with longer follow-up periods to evaluate treatment durability and recurrence rates in OLP management. In conclusion, the present study underscores the importance of exploring alternative treatment options for OLP and emphasizes the need for personalized therapeutic approaches to improve patient outcomes and quality of life.
